# Multidimensional scaling for large genomic data sets

**DOI:** 10.1186/1471-2105-9-179

**Published:** 2008-04-04

**Authors:** Jengnan Tzeng, Henry Horng-Shing Lu, Wen-Hsiung Li

**Affiliations:** 1 Genomics Research Center, Academia Sinica, Taipei, 115 Taiwan.; 2 Institute of Statistics, National Chiao Tung University, 1001 Ta Hsueh Road, Hsinchu 30050, Taiwan.; 3 Department of Ecology and Evolution, University of Chicago, 1101 East 57^th ^Street, Chicago, IL, 60637 USA.

## Abstract

**Background:**

Multi-dimensional scaling (MDS) is aimed to represent high dimensional data in a low dimensional space with preservation of the similarities between data points. This reduction in dimensionality is crucial for analyzing and revealing the genuine structure hidden in the data. For noisy data, dimension reduction can effectively reduce the effect of noise on the embedded structure. For large data set, dimension reduction can effectively reduce information retrieval complexity. Thus, MDS techniques are used in many applications of data mining and gene network research. However, although there have been a number of studies that applied MDS techniques to genomics research, the number of analyzed data points was restricted by the high computational complexity of MDS. In general, a non-metric MDS method is faster than a metric MDS, but it does not preserve the true relationships. The computational complexity of most metric MDS methods is over *O(N*^2^*)*, so that it is difficult to process a data set of a large number of genes *N*, such as in the case of whole genome microarray data.

**Results:**

We developed a new rapid metric MDS method with a low computational complexity, making metric MDS applicable for large data sets. Computer simulation showed that the new method of split-and-combine MDS (SC-MDS) is fast, accurate and efficient. Our empirical studies using microarray data on the yeast cell cycle showed that the performance of K-means in the reduced dimensional space is similar to or slightly better than that of K-means in the original space, but about three times faster to obtain the clustering results. Our clustering results using SC-MDS are more stable than those in the original space. Hence, the proposed SC-MDS is useful for analyzing whole genome data.

**Conclusion:**

Our new method reduces the computational complexity from *O*(*N*^3^) to *O*(*N*) when the dimension of the feature space is far less than the number of genes *N*, and it successfully reconstructs the low dimensional representation as does the classical MDS. Its performance depends on the grouping method and the minimal number of the intersection points between groups. Feasible methods for grouping methods are suggested; each group must contain both neighboring and far apart data points. Our method can represent high dimensional large data set in a low dimensional space not only efficiently but also effectively.

## Background

Representing high dimensional data in a low dimensional space is an important task because it becomes much easier to study the information structure when the dimension is greatly reduced. The main idea of MDS techniques is to configure the coordinates of the data in the significant space such that the pairwise relationship of relocated data in a low dimensional space is similar to that in the high dimensional space of the original data. With the dimensional reduction, one can cluster the data relationships by their distribution in the low dimensional space and explore significant patterns. When the data configuration is Euclidean, MDS is similar to principle component analysis (PCA), which can remove inherent noise with its compact representation of data [1]. When the data configuration is nonlinear, MDS can be further improved to capture the imbedded manifold in data [2].

MDS techniques have been applied to many fields, e.g., pattern recognition, stock market analysis, and molecular conformational analysis. However, the computational complexity of most metric MDSs is over *O*(*N*^2^), though some non-metric methods can reduce the complexity to $O(N/\sqrt{N})$[3]. Genomics research represents a challenging application of MDS. Data from microarray experiments are typically noisy with a large number of genes, but few replicates and frequent data updates. Due to the high computational complexity, it is very difficult to apply MDS to whole genome data, such as ~6000 genes in yeast, not to mention ~23,000 genes in human.

Taguchi and Oono [4] developed a novel algorithm for non-metric MDS analysis and applied it to analyze patterns of gene expression. However, the result of a non-metric MDS method depends heavily on the initial configuration and non-metric MDS only preserves the order of similarities instead of the original scale of similarities. Therefore, it remains an important issue to reduce the computational complexity for a metric MDS. In this paper, we develop a fast metric MDS method for large data sets that is suitable for data analysis and updates. Indeed, the computational time for 6000 target data points is within 30 seconds in a PC with CPU 1.67 GHz and 2G memory.

We review typical MDS techniques in the following and propose a new MDS method to solve the problem of large data sets in Section 3. We split the data into overlapping subsets, apply our MDS technique to each subset, and then recombine the configurations of the subsets into the same space. We call this method the split-and-combine MDS (SC-MDS) method. The complexity of SC-MDS is *O*(*p*^2 ^*N*), where p is the dimension of the feature space, which is far smaller than the number of data points *N*. In Section 4, we evaluate the performance of SC-MDS using simulation, apply SC-MDS to the GO database, and lastly, improve the K-means clustering of gene expression profiles by applying SC-MDS to yeast cell cycle microarray data [5].

There are many different categories of MDS techniques. For example, a distinction can be made between metric and non-metric MDSs, between weighted and unweighted MDSs, between single matrix and multiple matrices, and between deterministic and probabilistic matrices [3,6]. In this section, we introduce three typical MDS methods that are relevant to the present work.

### Classical MDS (CMDS)

Torgerson [7] proposed the first MDS method. The distance is the Euclidian distance, and the similarity matrix is complete (with no missing data) and symmetric. The main idea was that given the Euclidean distances or the inner products among points, it is possible to construct a matrix *X *of Cartesian coordinates of these points in the Euclidean space. Torgerson derived matrix *X *from the distance (or similarity) matrix *D *and showed what to do when the distance matrix includes noisy data. The key is to apply the double centering operator and singular value decomposition (SVD).

Double centering is the process of subtracting the row and column means of a matrix from its elements and adding the grand mean. For example, suppose that *D *is a product matrix, that is, *D *= *X*^*T *^*X*, where *X *is a *p *× *N *matrix with each column of *X *being a vector in a *p *-dimension space. We define i as a *N *× 1 vector in which every element is one and $B={\left(X-\frac{1}{N}X\dot{1}{\dot{1}}^{T}\right)}^{T}(X-\frac{1}{N}X\dot{1}{\dot{1}}^{T})$ as the similarity matrix with the means of the column vectors being zero. Then we have

(1) $$\begin{array}{l} B={\left(X-\frac{1}{N}X\dot{1}{\dot{1}}^{T}\right)}^{T}(X-\frac{1}{N}X\dot{1}{\dot{1}}^{T})\\ =X^{T}X-\frac{1}{N}X^{T}X\dot{1}{\dot{1}}^{T}-\frac{1}{N}\dot{1}{\dot{1}}^{T}X^{T}X+\frac{1}{N^{2}}{\dot{\text{1}}\dot{\text{1}}}^{T}X^{T}X\dot{1}{\dot{1}}^{T}\\ =D-\frac{1}{N}D\dot{1}{\dot{1}}^{T}-\frac{1}{N}{\dot{\text{1}}\dot{\text{1}}}^{T}D+\frac{1}{N^{2}}\dot{1}{\dot{1}}^{T}D\dot{1}{\dot{1}}^{T}\\ =D-{\bar{D}}_{r}-{\bar{D}}_{c}+{\bar{D}}_{g}, \end{array}$$

where ${\bar{D}}_{r}$ denotes the row means, ${\bar{D}}_{c}$ denotes the column means and ${\bar{D}}_{g}$ denotes the grand mean. If $H=1-\frac{1}{N}{\dot{\text{1}}\dot{\text{1}}}^{T}$, (1) can be simplified as

(2) *B *= *HDH*.

After performing double centering on *D*, one can apply SVD to *B*. Since *B *is symmetric, the decomposition is of the form

(3) *B *= *UVU*^*T*^.

Hence, $\sqrt{B}=X-\frac{1}{N}X11^{T}=UV^{\frac{1}{2}}$, where the columns of $\sqrt{B}$ are the coordinates of data, with the mean of the data being moved to the original point.

When *D *is a distance matrix, $d_{i,j}=\sqrt{{\left(x_{i}-x_{j}\right)}^{T}(x_{i}-x_{j})}$, where *x*_*i *_is the configuration of the *i*-th data point. The double centering of *D*^2 ^is equal to -2*B*, provided that $\sum_{i=1}^{N}x_{i}=0$. Hence, the CMDS method performs double centering on *D*^2^, multiplies by $-\frac{1}{2}$, and then performs SVD, which gives the configurations of the data. Thus, the key scheme of PCA is embedded in this CMDS method. When we want to find out the *r *dimensional configurations of the data in the space generated by the *r *principal components, we only use the first leading *r *spectrums and *r *columns of *U *to generate *X*, that is,

(4) $$X=\sqrt{V_{r}}U_{r},$$

where *V*_*r *_is the *r *× *r *sub-matrix of *V *and *U*_*r *_is a matrix of size *N *× *r*.

There are many drawbacks of this method [8]. For example, missing data is not allowed and the computational complexity is *O*(*N*^3^). Hence, this method is not suitable for massive data sets.

### Chalmer's Linear Iteration Algorithm

One of the force-based models of MDS is the spring model [9]. It considers each point of the data as a vertex in a low dimensional space, with springs connecting each vertex and the distance (or the spring length) between vertices proportional to their high dimensional distance. If *d*_*i*, *j *_denotes the high dimensional distance between vertices *i *and *j*, and *δ*_*i*, *j *_denotes the low dimensional distance, then the stress between vertices *i *and *j *is proportional to |*d*_*i*,*j *_- *δ*_*i*, *j*_|. The spring model computes (*N *- 1) forces at each vertex per iteration, and the computational complexity of this model is *O*(*N*^2^) per iteration.

Chalmers [8] proposed a linear iteration time layout algorithm. Instead of computing all the forces at each point, he computed only constant forces in the neighborhood of each point and randomly chose another constant point that is not in the neighborhood to compute the large distance effect. Only constant points are computed at each point, so that the computational complexity is reduced to *O*(*N*) per iteration. This spring model does not find the steady state solution in general. One can only process a fixed number of iterations, say 8 or 10, as opposed to finding the converged solution. Note that the constant forces are selected in both the neighborhood and afar. Failure to incorporate one of these two forces will diminish the performance of this method [8].

### Anchor Point Method

As in Chalmers' linear layout algorithm, in the anchor point MDS method [10] only a portion of data is used to reconstruct the layout for intermediate steps. The data are grouped into clusters, so that the distances between points in different clusters are less meaningful than the distances between points in the same cluster.

In this method, some points in the same cluster are chosen as anchors and others are considered as floaters. Distance information of anchors is used to construct the coarse structure of layout, and the floaters are used to update the fine structure. When a small number of *K *anchor points are chosen, a modified MDS procedure only computes the *N *× *K *matrix. Buja *et al. *[10] showed that the number of anchors could not be smaller than the dimension *p *of the given data. Moreover, the anchors should be chosen carefully because random choices of anchors do not work [10]. This is challenging when the grouping structure is unknown.

From these two methods, we can see that the intermediate steps for calculating MDS do not need to employ all entries of the dissimilarity matrix. We can use this property to reduce the computational complexity of MDS. Another important issue is choosing the number of dimensions for layout. In a small data set, one can use the elbow test or similar methods to detect the changing shape for the decay of the spectrum of SVD to determine the layout dimension. In a large data set, one feasible approach is to use stochastic methods by cross-validation to measure the layout dimension [11].

## Methods

We first describe SC-MDS using a simple case, and for convenience we use the classical MDS (CMDS) as the default MDS method to show how we can improve it. Assume that *x*_*i *_∈ *R*^*p *^are the coordinates of data points for *i *= 1,...,*N *and *p *<<*N*. We define *d*_*i*,*j *_= ||*x*_*i *_- *x*_*j*_||_2 _as the Euclidean distance between *x*_*i *_and *x*_*j*_. *N *is large such that applying the CMDS technique is impractical. We split the points into two overlapping sets *S*_1 _and *S*_2_, and the intersection of these two sets contains more than *p *points. The main idea is to apply MDS to each individual set to get the configuration, and to use the information of the overlapping points to combine the two sets into one. There are two problems that need to be solved: (1) how to combine two sets into one and (2) what is the sufficient condition for this solution to be equivalent to that obtained by working directly with the full set? The solutions to these two problems are proposed in the next subsections.

### Combination Method

When we split the whole set of data points into two overlapping subsets of equal sizes, the combined size of the two distance matrices for the two subsets is less than that for the whole set. Assume that the configurations of these two subsets obtained from MDS are *x*_*i*,1_and *x*_*i*,2 _and the dimensions of the two configurations are the same. We can fix the coordinates of the data points in the first set and use the overlapping data points to find an affine mapping *U*(·) + *b *such that for each intersection point *x*_*k*,1 _∈ *S*_1_, *x*_*k*,1 _= *Ux*_*j*,2 _+ *b*, where *x*_*j*,2 _∈ *S*_2 _for some *j*. Note that the matrix U of the affine mapping is a unitary matrix, which is a volume-preserving operator. The affine mapping can be found as follows.

Assume *X*_1 _and *X*_2 _are matrices in which the columns are the two coordinates of the overlapping points obtained by applying MDS to two data sets, and ${\bar{X}}_{1}$ and ${\bar{X}}_{2}$ are the means of columns of *X*_1 _and *X*_2_, respectively. In order to use the same orthogonal basis to represent these vertices, we apply QR factorization to $X_{1}-{\bar{X}}_{1}{\dot{1}}^{T}$ and $X_{2}-{\bar{X}}_{2}{\dot{1}}^{T}$, so that $X_{1}-{\bar{X}}_{1}{\dot{1}}^{T}=Q_{1}R_{1}$ and $X_{2}-{\bar{X}}_{2}{\dot{1}}^{T}=Q_{2}R_{2}$. Since these two coordinates represent the same points, the triangular matrices *R*_1 _and *R*_2 _should be identical when there is no rounding error from computing the QR factorization in *X*_1 _and *X*_2_. The positive and negative signs of columns of *Q*_*i *_could be arbitrarily assigned in the computation of QR factorization. Hence, the signs of columns of *Q*_*i *_should be adjusted according to the corresponding diagonal elements of *R*_*i *_so that the signs of diagonal elements of *R*_1 _and *R*_2 _become the same.

After the signs of columns of *Q*_*i *_are modified, we conclude

(5) $$Q_{1}^{T}(X_{1}-{\bar{X}}_{1}{\dot{1}}^{T})=Q_{2}^{T}(X_{2}-{\bar{X}}_{2}{\dot{1}}^{T}).$$

Furthermore, we can obtain

(6) $$X_{1}=Q_{1}Q_{2}^{T}X_{2}-Q_{1}Q_{2}^{T}({\bar{X}}_{2}{\dot{1}}^{T})+{\bar{X}}_{1}{\dot{1}}^{T}.$$

That is, the unitary operator is $U=Q_{1}Q_{2}^{T}$ and the shifting operator is $b=-Q_{1}Q_{2}^{T}{\bar{X}}_{2}+{\bar{X}}_{1}$.

In practice, one problem with the *X*_1 _and *X*_2 _obtained by CMDS from a distance matrix is that some of the eigenvalues of the double centered distance matrix can be negative. When negative eigenvalues occur, the dimension of the configuration is determined by the number of positive eigenvalues. This could cause those two triangular matrices *R*_1 _and *R*_2 _to be unequal and sometimes the dimensions of the configurations of the two subsets are different. In the case of equal dimensions, we can still use equation (6) to combine the two data sets into one, but the equality in equation (6) becomes an approximation and the combination will induce computing errors. In the case that the dimension of *X*_1 _is not equal to that of *X*_2_, for example, dim(*S*_1_) = *q*_1 _< dim(*S*_2_) = *q*_2_, we project *x*_*i*,2 _into the space generated by the leading *q*_1 _basis of *Q*_2 _We then use the new projected configuration of *x*_*i*,2 _and the configuration *x*_*i*,1 _to perform the combination processing. The projection of *x*_*i*,2 _from *q*_2 _dimension to *q*_1 _dimension induces computational errors too. To avoid this error, the sample number of the overlapping region is important. This sample number must be large enough so that the derived dimension of data is greater or equal to the real data.

### Sufficient Condition for Successive Combinations

In the case of a large number of data points, the data points are split into several overlapping groups such that the number of overlapping points is greater than the dimension of real data. The recombination approach is similar to the case of two overlapping subsets. For example, we split data points into *K *overlapping chained subsets {*S*_1_,...,*S*_*k*_}, i.e. *S*_*i *_∩ *S*_*i*+1 _≠ ∅; we apply the MDS techniques to each *S*_*i*_; we use the configurations of *S*_1 _as the central reference and combine the subsets around *S*_1_; we repeat this procedure until all the subsets are combined.

**Figure 1:**
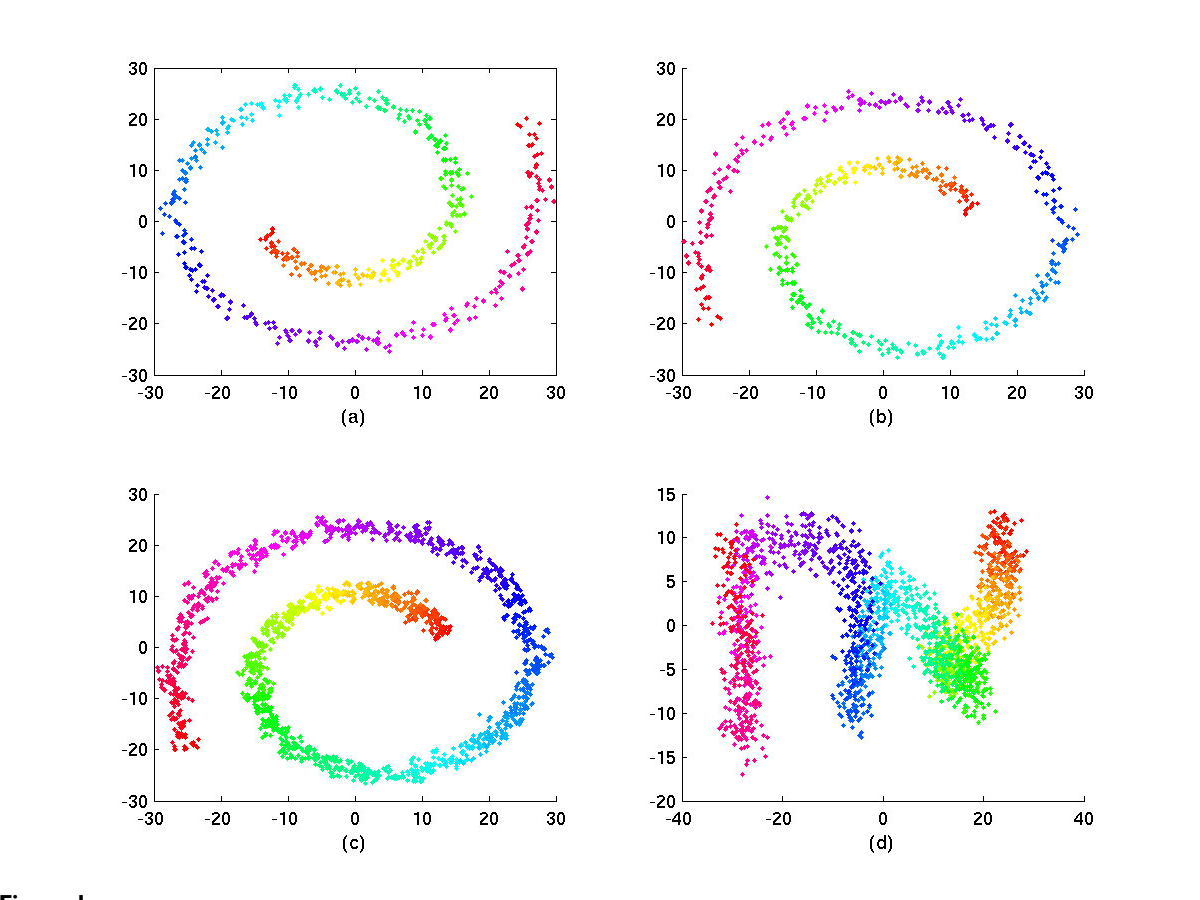
2D simulation results. a. The 2D CMDS projection of 2000 simulation points. b. The 2D layout of an approximate solution by SC-MDS with no dimension loss (*N*_*i *_= 30, *N*_*g *_= 200). The points in a group were chosen randomly. c. The 2D layout of the approximate solution by SC-MDS with a loss of 12 dimensions (*N*_*I *_= 5, *N*_*g *_= 200). The points in a group were chosen randomly. d. The 2D layout of the approximate solution by SC-MDS with a loss of 12 dimensions (*N*_*I *_= 5, *N*_*g *_= 200). The points in a group were chosen by the neighboring method.

The minimal number of points of each overlapping region and the grouping method used will strongly affect the performance of the low dimensional layout of MDS. Firstly, if the number of the overlapping points is smaller than the real data dimension, the rank of the affine mapping will be less than the dimension of data and the affine mapping cannot transform the coordinates to the corresponding coordinates. We demonstrate this point by a simulation case in the next section. Secondly, points of each group should be chosen both in the neighborhood and beyond. This has been mentioned in [3], where the information of both the neighborhood and afar is used for the spring model. If one puts only the neighboring points into the same group, the rotation effect will hamper the performance of the low dimensional layout (see Fig. 1d later). These two conditions are sufficient to guarantee good performance in a low dimension layout. The layout of the correct approximation solution of our method is outlined (see Fig. 1b).

### Computational Complexity Reduction

We now show how SC-MDS reduces the computational complexity of CMDS from *O*(*N*^3^) to *O*(*N*) when *p *<<*N*. Assume that there are *N *points in a data set, *N*_*I *_is the number of points in each intersection region, and *N*_*g *_is the number of points in each group. When we split *N *points into *K *overlapping groups, we have *KN*_*g *_- (*K*-1)*N*_*I *_= *N*, and we have $K=\frac{\left(N-N_{I}\right)}{(N_{g}-N_{I}}\sim O(N)$.

For each group, we apply CMDS to compute the coordinates of the group data, which costs $O(N_{g}^{3})$ computation time. At each overlapping region, we apply QR factorization to compute the affine transform, which costs $O(N_{I}^{3})$ computation time. Since the lower bound of *N*_*I *_is *p*+1, we can assume that *N*_*g *_= *αp *for some constant *α*. Then the total computation time is about

(7) $$\frac{N-p}{(\alpha -1)p}O(\alpha^{3}p^{3})+\frac{N-\alpha p}{(\alpha -1)p}O(p^{3})\sim O(p^{2}N).$$

Thus, when *p *<<*N*, the computation time of the SC-MDS becomes *O*(*p*^2^*N*). Moreover, if $p<\sqrt{N}$, the computational complexity is smaller than $O(\sqrt{N}N)$, which is the computational complexity for the fast MDS method proposed by Morrison *et al*. [12]. When *p *is very large (say *p*^2 ^> *N*), SC-MDS has no advantage in computational speed, but it makes the computation feasible even when the computer memory does not afford the MDS computation. Furthermore, if we do not use CMDS as our default MDS method but use the linear time algorithm [8] instead, then we can further reduce the complexity of the SC-MDS method to *O*(*pN*). This improvement makes application of the MDS to large data sets feasible. Hence, data analysis using SC-MDS can guarantee better accuracy than existing non-metric MDS methods.

## Results and Discussion

### Simulation experiments

We simulate a spiral in two dimensions:

*r *= 2*θ*, 2*π *≤ *θ *≤ 5*π*.

First, let us construct the reference coordinates *X*. Discretize *θ *into *N*-1 intervals and let *θ*_*i *_= 2*π *+ *idθ*, $d\theta =\frac{3\pi }{N-1}$. At each *θ*_*i*_, *k *points are constructed with noise. The following steps generate the data:

Construct two raw vectors *q*_1 _and *q*_2_,

$$\begin{array}{c} q_{1,i}=2\theta_{\left\lfloor i/k\right\rfloor }\cos\theta_{\left\lfloor i/k\right\rfloor }+n_{i},\\ q_{2,i}=2\theta_{\left\lfloor i/k\right\rfloor }\sin\theta_{\left\lfloor i/k\right\rfloor }+n_{i}, \end{array}$$

where *n*_*i *_is a random variable with normal distribution *n*_*i *_~ *N*(0,1), for *i *= 1⋯*kN*. Then we add some *p*-2 dimensional randomness into the coordinate matrix as follows. Let *Z *= ⟨*α*_*j*_*u*_*i*,*j*_⟩, *u*_*i*,*j *_~ *N*(0,1), for *i *= 1⋯*kN *and *j *= 1⋯*p *- 2, where *α*_*j *_is the parameter to control the standard deviation of random variables. The final reference coordinates matrix *X *is

*X *= [*q*_1_, *q*_2_, *Z*]^*T *^= [*x*_1_, *x*_2_,...,*x*_*kN*_],

where *x*_*i *_∈ *R*^*p*^. So the distance matrix is *D *= ⟨*d*_*i*, *j*_⟩ with *d*_*i*,*j *_= ||*x*_*i *_- *x*_*j*_||. In this simulation, *p *= 17 is used.

**Figure 2:**
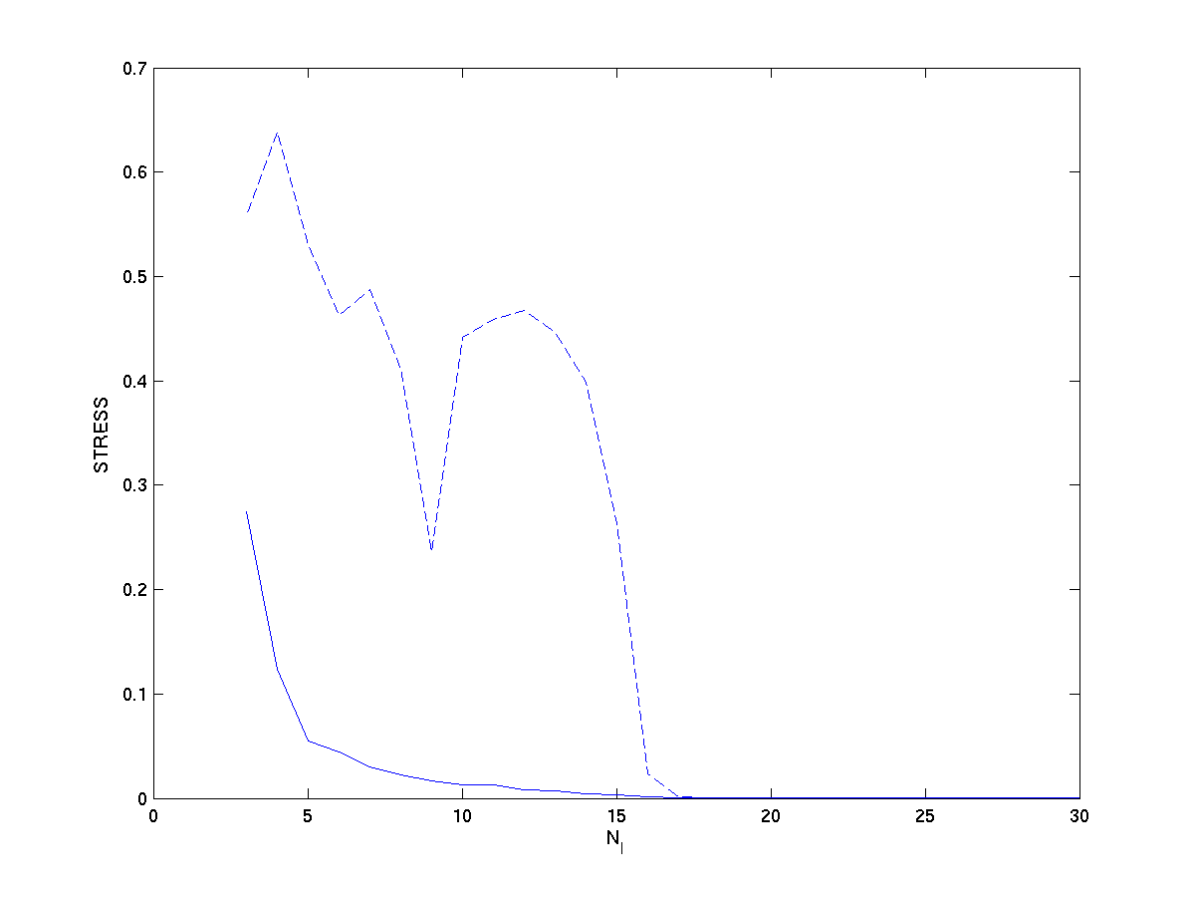
Computational error as a function of *N*_*I*_. The solid line refers to the case where the points in each group are chosen randomly, while the dash line refers to the case where the points in each group are chosen from neighbors. In all cases, *N*_*g *_is fixed to 200.

Fig. 1a is the 2D projection obtained by applying CMDS to 2000 simulation points. Then, in the first application of SC-MDS, we set the number of points in each group to *N*_*g *_= 200 and the number of points in each intersection region to *N*_*I *_= 30 such that the number of the overlapping points is greater than the real dimension 17. All points in each group are chosen randomly. In order to measure the difference between classical MDS and SC-MDS, we use the STRESS (Kruskal's goodness of fit index) to compute the error between the distance matrixes. The formula of STRESS is

$$STRESS=\sqrt{\frac{\sum_{i,j}{\left(d_{i,j}-{\tilde{d}}_{i,j}\right)}^{2}}{\sum_{i,j}d_{i,j}^{2}}},$$

where *d*_*i*,*j *_refers to the distance matrix of the original data and ${\tilde{d}}_{i,j}$ refers to that for the SC-MDS reconstruction. In this spiral example, the STRESS of computing errors for SC-MDS is only 3.93 × 10^-14^, and the STRESS for CMDS is only 1.25 × 10^-15^. These errors are here considered as rounding errors. Thus, SC-MDS can reconstruct the configuration as does CMDS; the result of our method (Fig 1b) is similar to the 2D projection in Fig. 1a. Because this spiral example has only two essential dimensions, the 3rd to 17th dimensions are considered as random perturbations. If we reduce the number *N*_*I *_from 16 to 3 but keep *N*_*g *_= 200, we can see that the 2D SC-MDS representation keeps the shape of spiral but becomes more and more blurred as *N*_*I *_decreases. At the same time, the STRESS increases when *N*_*I *_decreases, as will be shown in Fig 2. In Fig. 1c, we set *N*_*g *_= 200 and *N*_*I *_= 5 and the points in each group are also chosen randomly. The performance is now slightly worse than CMD due to dimension loss; the STRESS here is 0.0398. In Fig. 1d, we set *N*_*g *_= 200 and *N*_*I *_= 5, and group points neighboring to each other into the same group. It shows that the performance is totally bad – the spiral structure is lost and the STRESS increases to 0.5232.

Figure 2 shows the correlation between STRESS and the number of points in the overlapping region *N*_*I*_. There are two lines. The solid line refers to points in each group that are chosen randomly, while the dash line refers to points in each group that are chosen from neighbors; all *N*_*g *_are fixed to 200. Points in each line are the average of 20 repeats of STRESS computed with different randomly choosing points in each group. We can see that the STRESS of each line decays as *N*_*I *_increases. When *N*_*I *_is larger than the real dimension of data, the STRESS is almost zero. When *N*_*I *_is smaller than the real dimension of data, the performance of solid line is better than the dash line. Without the afar information in each group, the performance is worse. That is, randomly choosing points in each group helps to reduce the error when *N*_*I *_is not large enough.

In the same simulation example, we observed the relationship between error and the number of groups. When *N*_*I *_is larger than the dimension of the data, like *N*_*I *_= 20, we can reconstruct the configuration of the data well. The number of groups of our grouping method does not affect the STRESS and the average STRESS is ~10^-12^. Hence, SC-MDS gives accurate results if we carefully control the number of overlapping points and choose random points in each group.

Next, we compare the speeds of non-metric MDS, CMDS and SC-MDS. We create random vectors in a 20-dimension space, with the sample size ranging from 50 to 2000.

**Figure 3:**
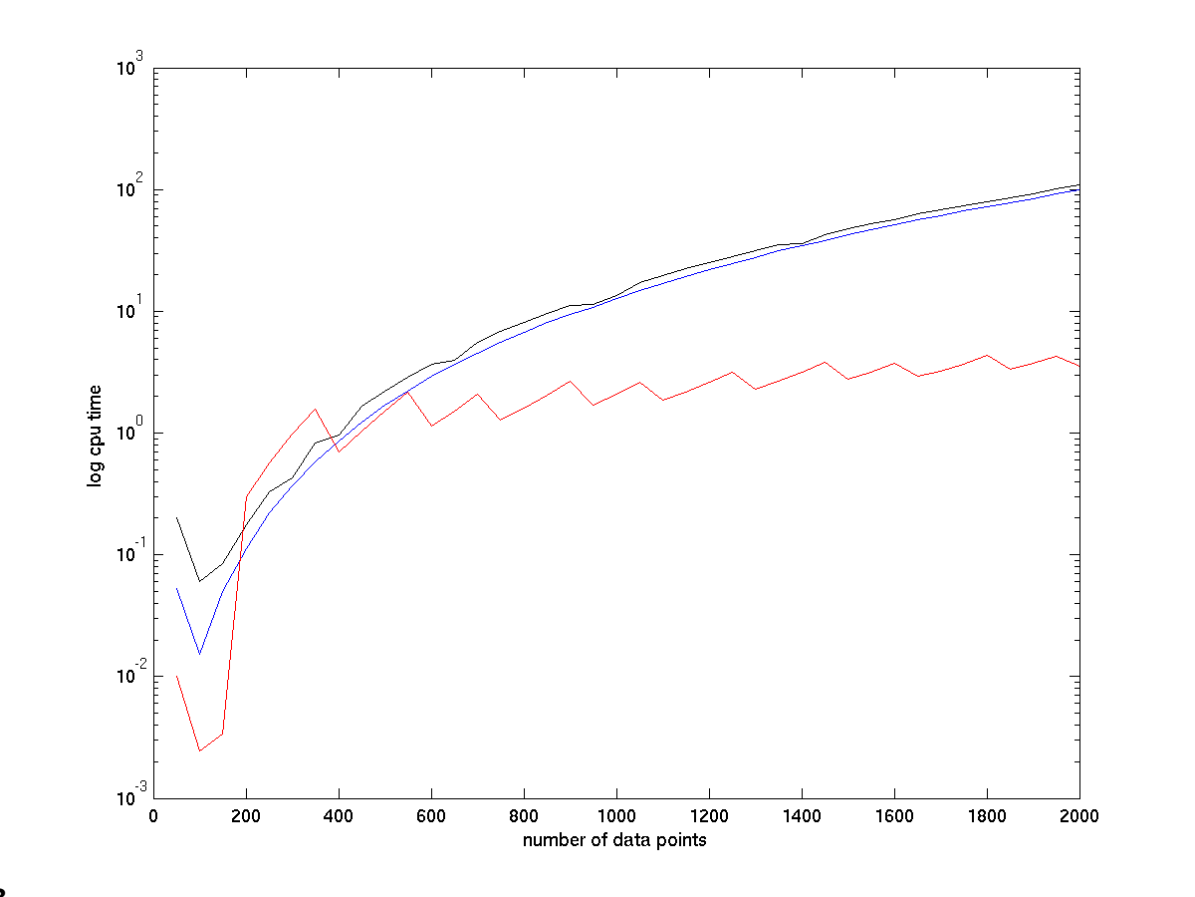
Speed comparison among non-metric MDS, CMDS and SC-MDS. The SC-MDS method is denoted by the red line, the CMDS method is denoted by the blue line, and the non-metric MDS method is denoted by the black line. The black and red lines are produced by average of 20 repeats.

In Figure 3 the black line (non-metric MDS) and the blue line (CMDS) are produced by Matlab default scripts, mdscale.m and cmdscale.m. The red line refers to our SC-MDS method. We can see that the simulation result matches our theoretical analysis.

Because of the hardware limitation to process CMDS, we use only a set of 2000 sample points as the maximal sample size to demonstrate that SC-MDS performs as well as CMDS when the number of data points (N) is not very large. We give below an example of large N to show that SC-MDS works well in a large data set that cannot be handled by CMDS.

### Gene Correlation Map

Gene correlation maps are used to represent the correlations of genes such that genes with similar biological functions or in the same biological pathway tend to be located in the same neighborhood. It provides a prior probability in many applications of genome research by Bayesian methods. Since Affy U133A GeneChip is widely used in many studies, we used genes listed in this chip and GO descriptions on the Gene Ontology website to create the gene correlation map. In this chip, there are 22,283 genes and 2168 GO terms are used in the list of these genes. Hence, we consider each gene a binary vector with length of 2168. If the *i*-th term of the vector is one, then this gene has the *i*-th GO description. There are 5781 genes without any GO description so that these genes are not used to compute the correlation with the genes with a GO description. Hence, the term-document matrix is reduced to the size of 16502 × 2168.

**Figure 4:**
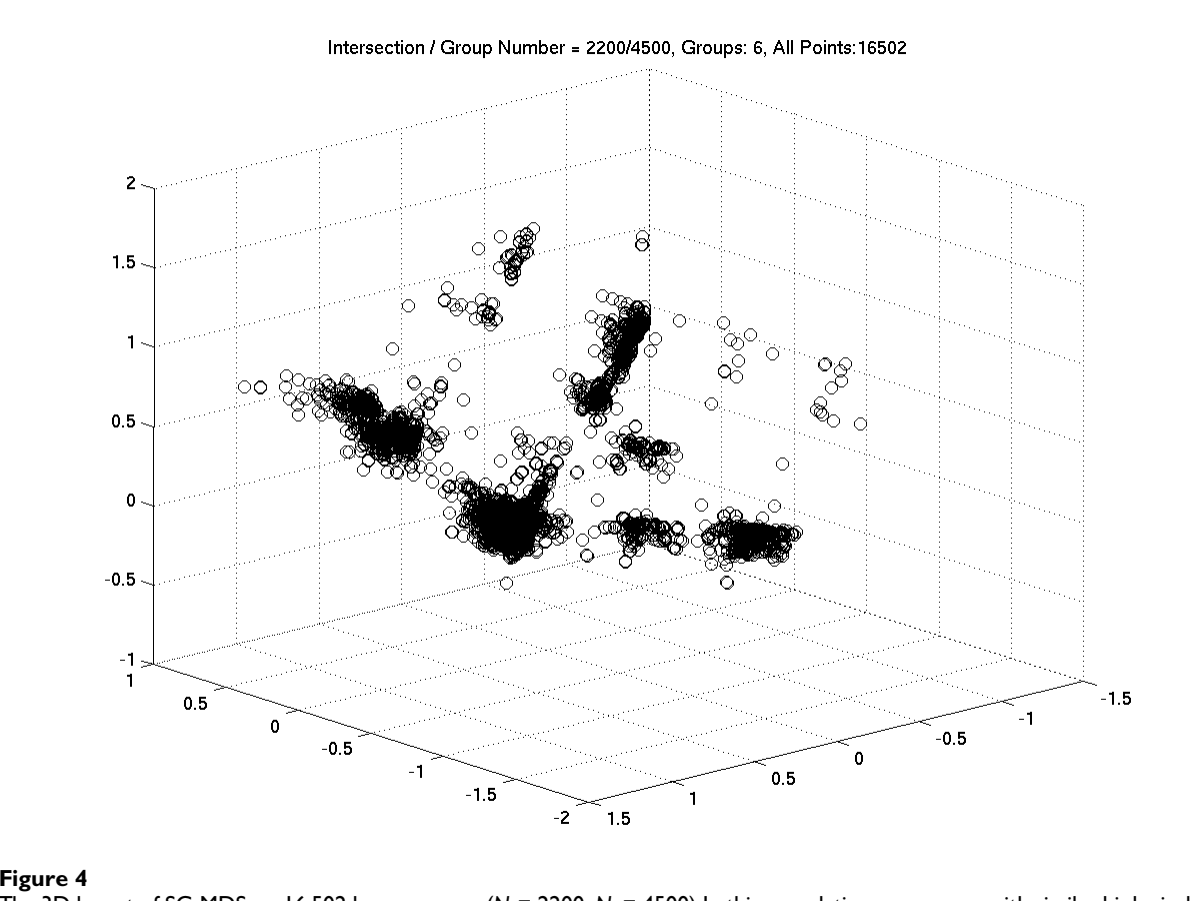
The 3D layout of SC-MDS on 16,502 human genes. (*N*_*I *_= 2200, *N*_*g *_= 4500) In this correlation map, genes with similar biological functions or in the same biological pathway tend to be located in the same neighborhood.

To apply the CMDS to a distance matrix of 16502 × 16502 is impossible for current hardware. So we use SC-MDS to randomly separate the 16,502 genes into 6 overlapping subsets, where *N*_*I *_= 2200 and *N*_*g *_= 4500. Although the essential dimension should be smaller then 2168, we still use *N*_*I *_= 2200 to ensure accurate reconstruction. The QR operation and SVD operation are available for this size. In each subset, we compute the 4500 × 4500 distance matrix and then compute the 3D MDS layout. Figure 4 is the 3D layout of SC-MDS results on these 16,502 genes. In this figure, two genes located closely have similar GO descriptions. We use the Euclidian distance in this 3D layout to measure the relationship between genes. For example, gene probe IDs 220259_at, 220815_at, 221980_at, 201015_s_at, 209880_s_at, 219765_at, 203018_s_at, 205523_at, 209879_at, 218796_at refer to the same GO description and they have the same coordinates in Figure 4. This gene correlation map is useful for many gene selection problems. Although we could not validate this result by comparing it with CMDS, we can repeat the SC-MDS procedure to see if we get consistent results among repeats. We find that the values of STRESS between different repeats are all small. This suggests that our method is stable for this large data set.

**Figure 5:**
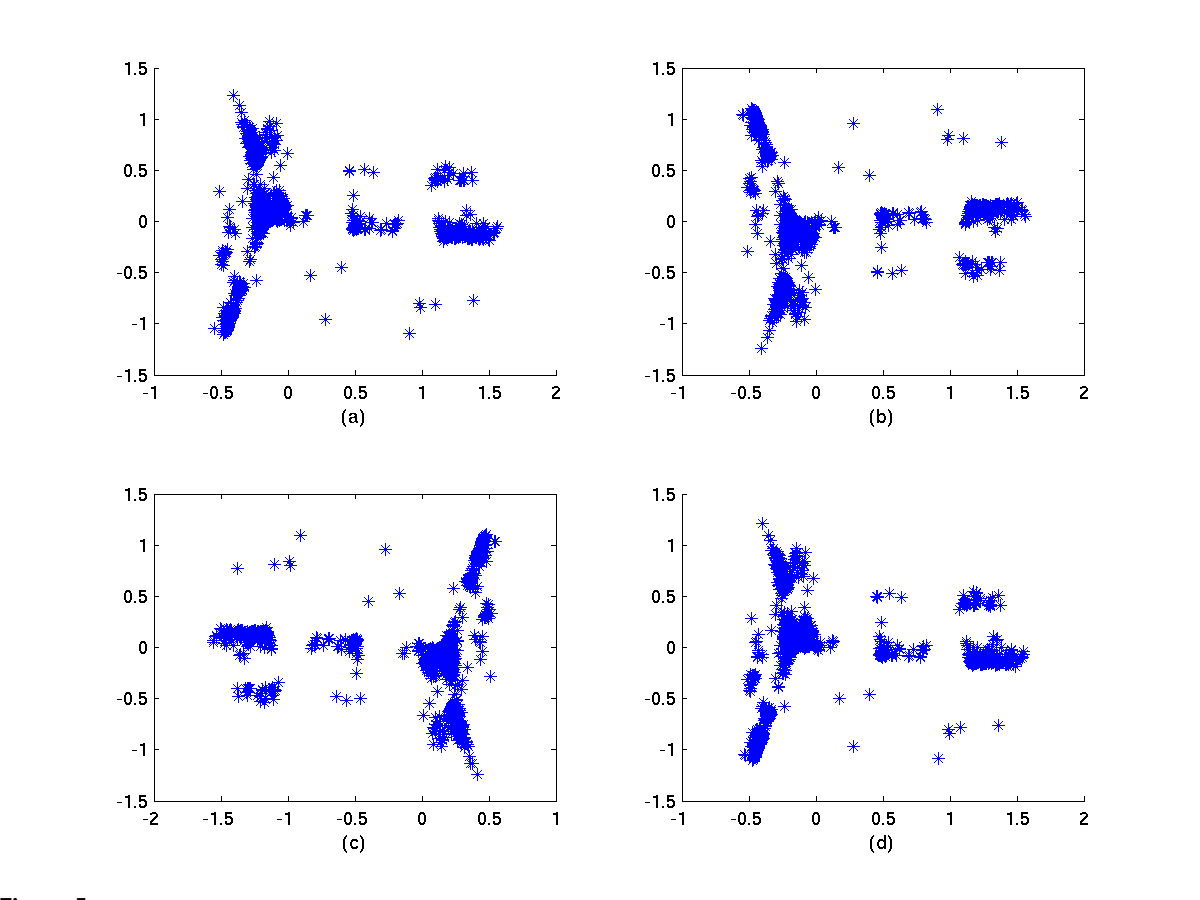
2D MDS comparison of 4000 GO samples. a The 2D CMDS projection of 4000 sampled genes of Affy U133A GeneChip. b The 2D layout of an approximate solution by SC-MDS with no dimension loss (*N*_*I *_= 2200, *N*_*g *_= 2500). The points in a group are chosen *g*randomly. c The 2D layout of the approximate solution by SC-MDS with loss of 1058 dimensions (*N*_*I *_= 1110, *N*_*g *_= 2000). The points in a group are chosen randomly. d The 2D layout of the approximate solution by SC-MDS with loss of 1668 dimensions (*N*_*I *_= 500, *N*_*g *_= 1000). The points in a group are chosen randomly.

Another way to check our method is to sample a subset of genes to create the correlation map, for example, 4000 sampled genes. Then we can compare the performance between CMDS and SC-MDS. We randomly sampled 4000 genes from the 16,502 genes, and then compute the distance matrix corresponding to these genes. The CMDS layout showed that there are 1106 dimensions in this gene set. Then we applied SC-MDS to this distance matrix with three different pairs (*N*_*I *_= 2200, *N*_*g *_= 2500), (*N*_*I *_= 1110, *N*_*g *_= 2000) and (*N*_*I *_= 500, *N*_*g *_= 1000). We repeated SC-MDS 10 times for each pair. Figure 5a is the 2D CMDS layout. The STRESS between the original data and CMDS is 1.796 × 10^-15^. Figure 5b is the 2D SC-MDS layout with (*N*_*I *_= 2200, *N*_*g *_= 2500), and its average STRESS compared with the original data is 0.0138. Figure 5c is the 2D SC-MDS layout with (*N*_*I *_= 1110, *N*_*g *_= 2000), its average STRESS is 0.0140. Figure 5d is 2D SC-MDS layout with (*N*_*I *_= 500, *N*_*g *_= 1000), and its average STRESS is 0.0282. We can see that all the 2D structures are preserved, though less well in Figure 5d where there is a substantial dimension loss.

### Gene expression clustering

The goal of gene expression clustering is to subdivide a set of gene expressing profiles into clusters such that genes in the same cluster share the same or similar patterns of expression profiles. In the situation with high dimensional data, researchers tend to obtain a manifold, which is defined by the regression of the data. Because gene expression data is typically noisy, by clustering the projection of data in this manifold, which is in a lower dimensional space in comparison to the original data, a better result can be obtained. In this section, we show that the SC-MDS method can successfully transform a high dimensional gene expression data set to a much lower dimension and preserve the intrinsic information of the original data. This transformation makes the clustering algorithm faster to get a converged solution. Moreover, the representation of these gene expression data in this lower dimensional space reveals a better clustering result in biological validation.

We use the α38-synchronized yeast cell cycle dataset [5]. There were 5006 genes in the data set, and each gene has a 25 point-time-course expression profile. However, this expression profile contained missing values, and we input these missing values by the KNN imputation method [13]. To avoid the synchronized block effect, we remove the first two points of the time course data, so that the expression profiles for each gene are left with 23 time-course points. We then (1) compute the pair-wise dissimilarity of genes from each subset that are randomly chosen by the sufficient condition of SC-MDS; (2) apply SC-MDS to the subsets to reconstruct the new coordinate in the feature space; and (3) cluster genes in this feature space. Because CMDS cannot handle large samples and thus cannot be used to reduce the dimension when the sample size is large, we do not consider the clustering results in the reduced dimension space derived from CMDS. Instead, we compare the clustering results in the reduced dimension space derived from SC-MDS with the results obtained in the original (non-reduced) space.

Using the standard Euclidean distance to measure the pair-wise dissimilarity of genes, we process the SC-MDS method such that the data points are split into 61 groups, *N*_*I *_= 100 and *N*_*g *_= 200.

Note that instead of computing the pair-wise dissimilarity for every pair, we need to compute only the pair-wise dissimilarity in each group. This reduces the computational complexity of all processing to *O(N)*. We choose *N*_*I *_to be greater than the length of original time course data points to satisfy the sufficient condition for an accurate layout.

**Figure 6:**
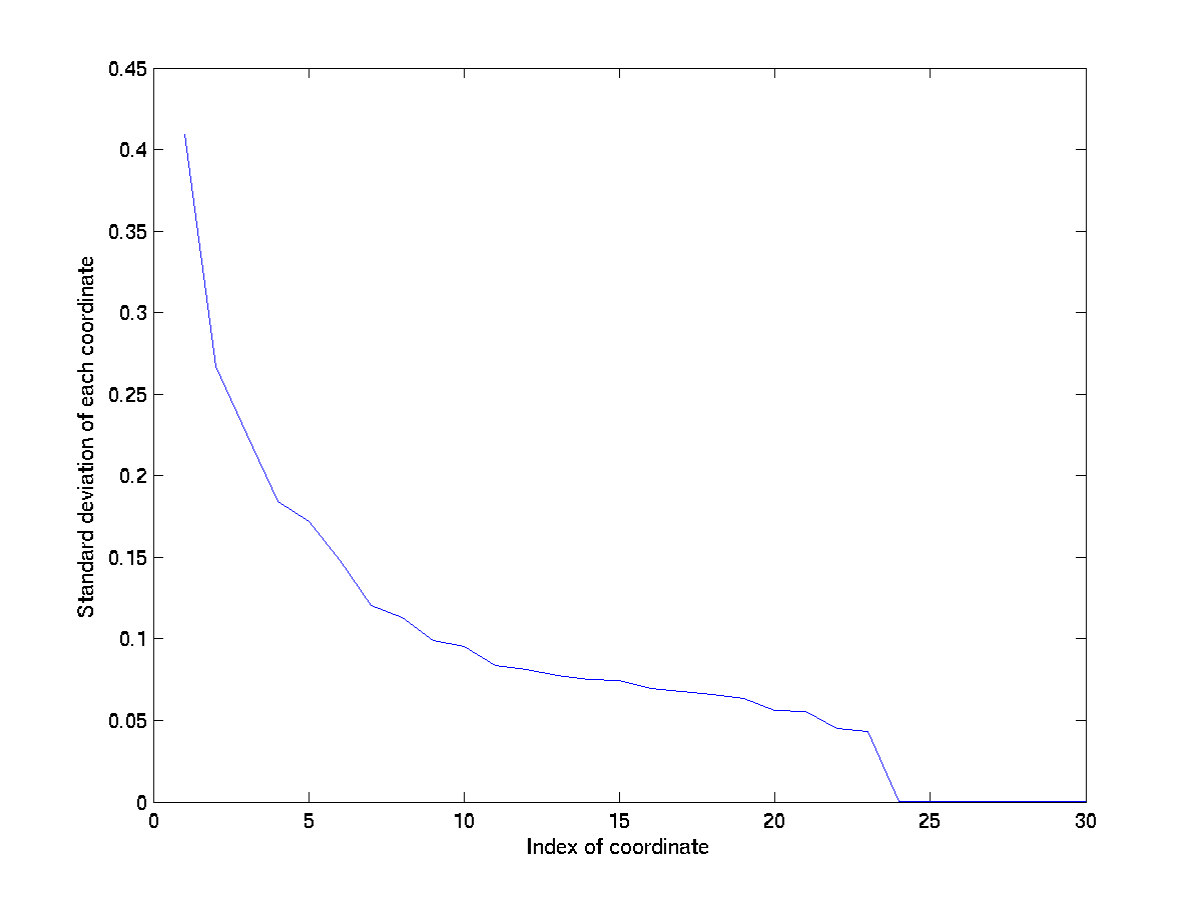
The standard deviation of each coordinate of the layout representation. The first turning point occurs at dimension 4, the second turning point occurs at dimension 7, and after dimension 11 the standard deviation decays smoothly.

Using a PC with CPU 1.67 GHz, 2G memory and Matlab R14 as the testing software, we complete the analysis with the CPU time of ~46 seconds. From the degradation rate of the distribution of standard deviations of output coordinates (Figure 6) the derived from the average of 30 repeats with different randomly grouping the elements of each subgroup, there are turning points at the 4^th^, 7^th ^and 11^th ^coordinates; after the 11th coordinate, the degradation is very small. By examining the second order difference of Figure 6, a local extremum occurs at 4^th^, 7^th ^and 11^th ^coordinates. Because the standard deviation decreases smoothly after the 7^th ^coordinate, and because the variation of the concavity in the 7^th ^coordinate is larger than those at the 4^th ^and 11^th ^coordinates, we assume that the variables after dimension 7 are noisy and can be removed for dimension reduction. Hence, we lay out these 5006 genes on a 7-dimensional space. In this space, two genes located nearby will have similar expression profiles in this dataset. Then we perform the K-means clustering method to compare the clustering result between the original expression data and our low dimensional layout.

**Figure 7:**
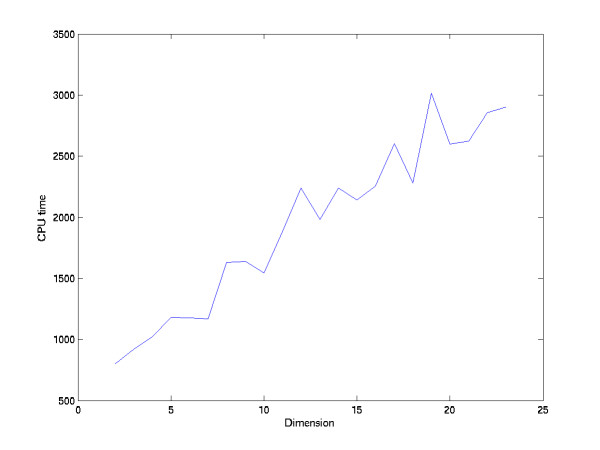
K-means convergence time vs. data dimension.

Note that K-means usually get a local optimal solution. When we want to obtain a reliable solution, we need to repeat K-means several times and choose the best solution. Or we can define a function to measure the quality of K-means solutions, and then apply the simulated annealing to get the optimal solution. Thus, obtaining a reliable K-means solution will take time. Fortunately, applying K-means in the reduced dimension space are more stable than applying K-means in the high dimension space. That is, the iteration of K-means in the reduced dimension space converges faster than in the high dimension space. To demonstrate how dimension affects the stability of K-means, we repeat K-means on the yeast cell cycle data in the SC-MDS space with different restricted dimensions, until K-means obtain 50 converged solutions. Figure 7 shows that the CPU times are inversely proportional to the data dimension. The time K-means requires in a seven dimensional space is ~1/3 of a 23 dimensional space. Hence dimension reduction accelerates the K-means solution. However, although applying K-means in a low dimension space is faster than in a high dimension space, loss of too many dimensions will distort the distance relationship between data points, thus distorting the clustering result. Hence, the dimension of reduced space should be determined carefully.

Before applying K-means clustering, we determine how many clusters we should use as the parameter in the K-means process. We search the number of clusters in the K-means process from 10 to 75. Since the clustering result of K-means depends on the initial guess of the centroids of the sets, we repeat 30 times the K-means process and use the Bayesian Information Criterion (BIC) score [14] to pick up the best clustering index from the mean of these 30 clustering results. In each K-means process, if it does not reach convergence in 100 iterations, we reset the initial values of the cluster centers and re-run the K-means process until the process converges. We set the upper bound for re-running time to be 5. If the re-running time reaches this upper bound, we choose the final cluster index by the best cluster sets from the previous iteration results.

**Figure 8:**
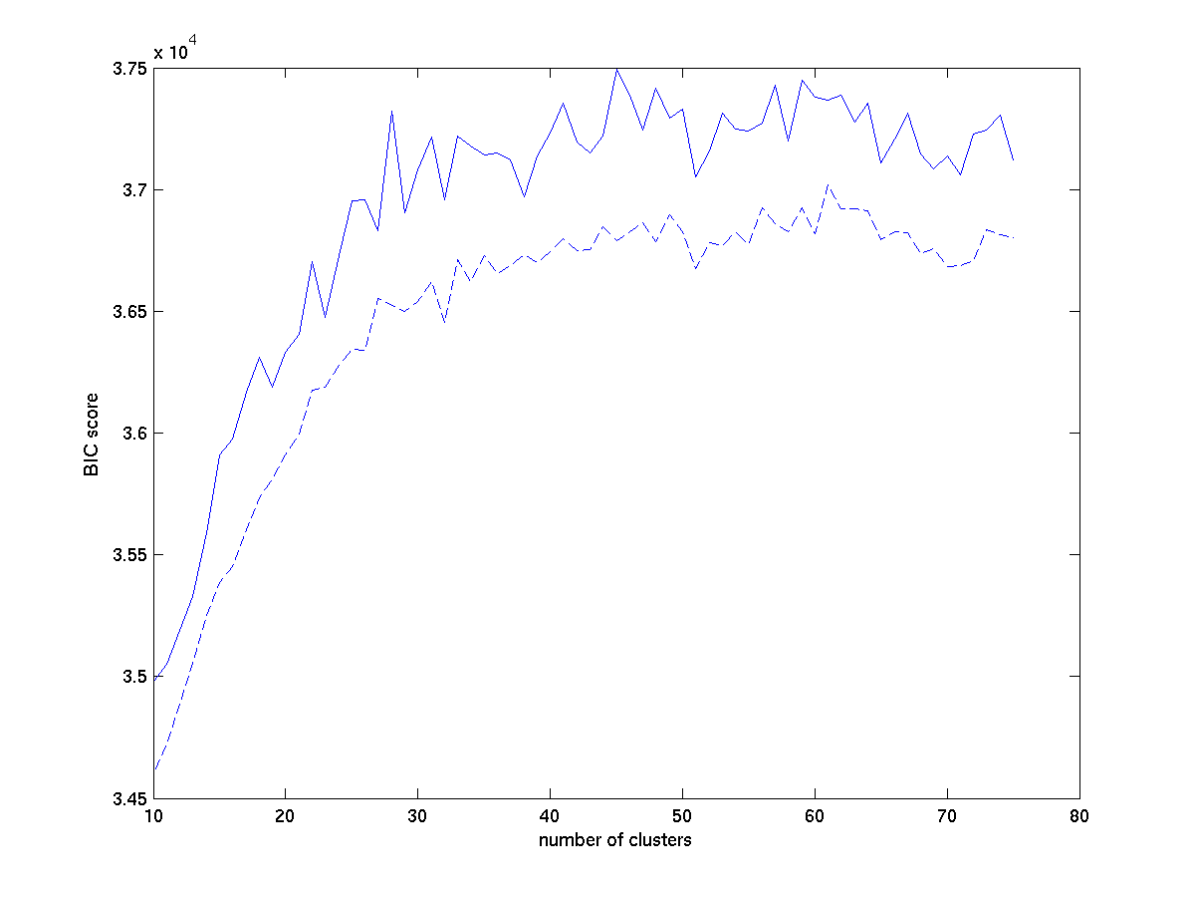
The curve of BIC Scores vs. number of clusters in K-means. The solid line denotes the max of 30 repeats of the BIC score. The dash line denotes the average of 30 repeats of the BIC score. The maximal value of the average curve occurs at 61 clusters. After 61 clusters the average curve starts decaying.

The BIC score is computed by the following formula:

$$BIC=\sum_{i=1}^{n}\log\left(\sum_{j=1}^{k}\mathrm{Pr}(x_{i}|c_{j})\mathrm{Pr}(c_{j})\right)-\frac{(k+1)p}{2}\log(n),$$

where *n *is the number of data points, *k *is the number of clusters, *c*_*j *_is the *j*-th model and *p *is the length of data points. We assume that each cluster from the K-means procedure is a multivariate normal distribution. The first term of the formula is the log-likelihood and the second term is penalty. When the data fit the model well, the term of log-likelihood becomes larger. We assume that the standard deviations of the multivariate normal distributions are the same, so that there are *(k+1)p *parameters, *k *means, and one standard deviation in the *p *dimensional space, in these *k *clusters (models). If a model becomes complicated, i.e., the number of the model parameters becomes large, the BIC score will decay. In Fig. 8, the dash line is reduced by taking the average of 30 repeats and the solid line is reduced by taking the maximal value of 30 repeats. We can see that the maximal value of solid line occurs in 45 clusters and the maximal of the dash line occurs in 61 clusters. Hence, we partition 5006 genes into 61 clusters in our example.

**Figure 9:**
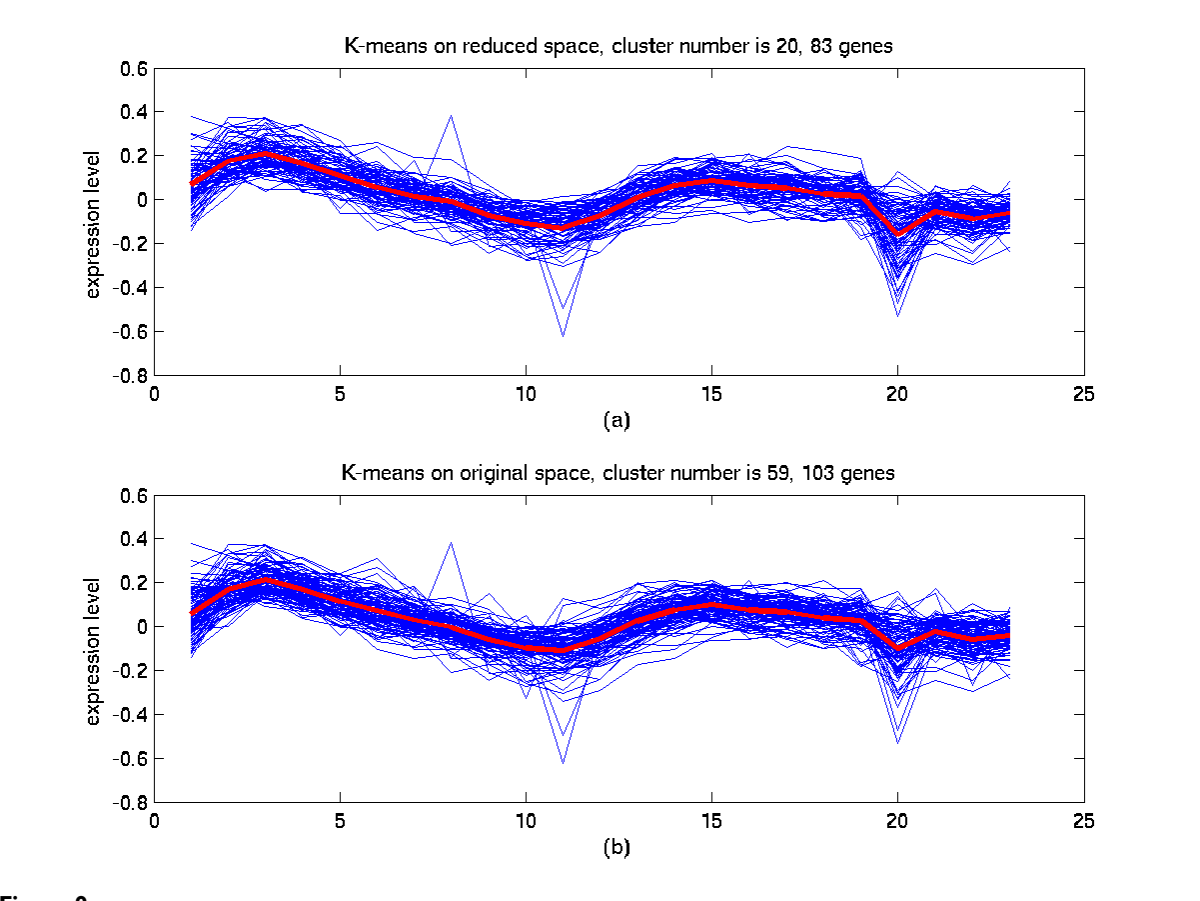
Comparison of the clustering results in the reduced space and in the original space (I). (a) The 20^th ^cluster of K-means in the 7-dimensional space from the SC-MDS method. 45.5% of the genes in this cluster are annotated cell cycle functions. (b) The 59^th ^cluster of K-means in the original 23-dimensional space. 40% of the genes in this cluster are annotated cell cycle functions. The blue lines are gene expression profiles and the red line indicates the average of these expression profiles.

**Figure 10:**
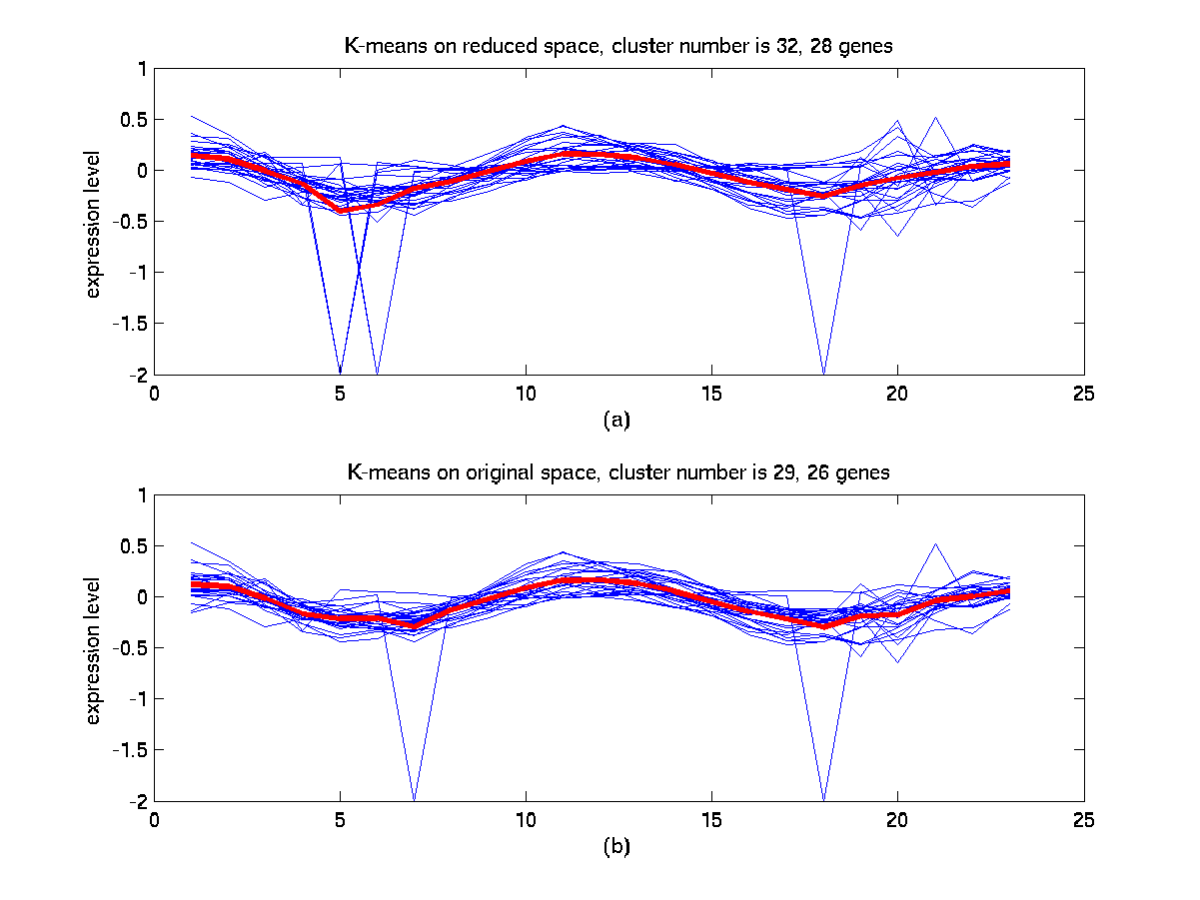
Comparison of the clustering results in the reduced space and in the original space (II). (a) The 32^th ^cluster of K-means in the 7-dimensional space from SC-MDS method. 51.8% of genes in this cluster are annotated cell cycle functions. (b) The 29^th ^cluster of K-means in the original 23-dimensional space. 42.3% of genes in this cluster are annotated cell cycle functions.

**Figure 11:**
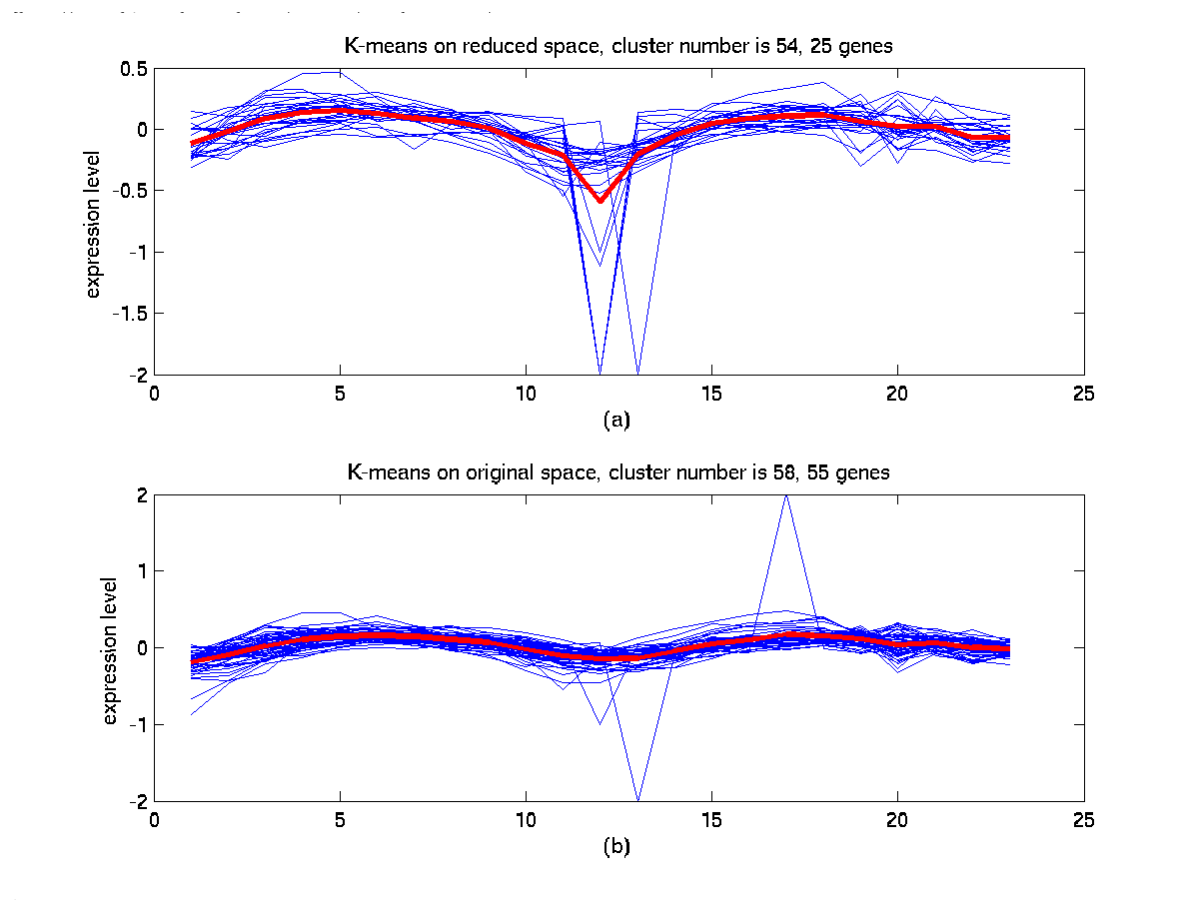
Comparison of the clustering results on the reduced space and on the original space (III). (a) The 54^th ^cluster of K-means in the 7-dimensional space from the SC-MDS method. 45.8% of genes in this cluster are annotated cell cycle functions. (b) The 58^th ^cluster of K-means in the original 23-dimensional space. 48% of genes in this cluster are annotated cell cycle functions.

We cluster the original data set and the reduced 7-dimensional space data set from SC-MDS separately, and each data set gives rise to 61 distinct gene clusters. Then we input gene names from these clusters to the MIPS Functional Catalogue Database. The outputs indicate that 13 clusters of the original clustering data set are significant to the cell cycle (*p*-value is smaller than 10^-3^). In contrast, 14 clusters of the 7-dimensional data set are significant to the cell cycle function. Twenty pairs of almost matched clusters, which consist of highly similar genes, are found by comparing the clusters from the two data sets. Nine clusters of the 7-dimensional data set are not annotated to any function, while 13 clusters of the original clustering data set are not annotated. The details of these paired clusters are shown in Additional file 1 and file 2. There are many unpaired clusters, which contain lower proportions (the match rate < 80%) of similar genes. In Figs. 9 to 11, we show these lower matched pairs that are related to cell cycle. In Figs. 9 and 10 the biological validation shows that these clusters obtained from SC-MDS are better than the corresponding clusters from the original 23-dimensional space data. In Fig. 9(a), 45.5% genes are indicated to have cell cycle function in the cluster obtained in the reduced space, and its p-value is 1.1 × 10^-9^. In Fig. 9(b), 40% genes are indicated to have cell cycle function in the cluster obtained in the original space, and its p-value is 1.4 × 10^-8^. There are, however, another 37 genes that are included in the corresponding cluster in the original space. Nine of them have significant functions in DNA processing. Conversely, there are another 17 genes that are included in the corresponding cluster in the reduced space. Six of them have significant function in mitotic cell cycle and cell cycle control. In Fig. 10, 51.8% of the genes in the 32^th ^cluster are annotated to have cell cycle functions in the cluster of K-means in the reduced space, while the genes in the corresponding cluster in the original space, the 29^th ^cluster, 42.3% are annotated to have cell cycle function. Compare the difference between Fig. 10(a) and 10(b). Five genes included in the cluster of the original space have no significant in the cell cycle function. Conversely, 7 genes included in the cluster of the reduced space include 3 genes (YHR152w, YML128c and YOR265w) that are significant in meiosis. In Fig. 11, 45.8% of the genes in the 54 cluster of the reduced space are annotated to have cell cycle, its p-value = 7.27 × 10^14^; 48% of the genes in the 58 cluster of the original space are annotated to cell cycle function, its p-value = 2.03 × 10^-7^. We can see that in this pair, the cluster in the reduced space is worse than that in the original space. The other comparisons between the clusters from the reduced 7-dimensional space data and from the original space data are shown in Additional file 1 and file 2. Combine the above three cases and twenty pairs that are almost matched, we can see that applying K-means to the SC-MDS reduced space performs at least as well as in the original data set. SC-MDS did not distort the pairwise relationship between data points, and it speeds up the analysis with accuracy preserved.

## Conclusion

Our new method reduces the computational complexity from *O*(*N*^3^) to *O*(*N*) when the dimension of the feature space is far less than the number of genes *N*, and it successfully reconstructs the low dimensional representation as does the classical MDS. Its performance depends on the grouping method and the minimal number of the intersection points between groups. Feasible methods for grouping methods are suggested; each group must contain both neighboring and far apart data points. Our method can represent a high dimensional large data set in a low dimensional space not only efficiently but also effectively. This Split-and-Combine method makes the parallel computation of MDS feasible. If samples increase to the level that one computer could not handle, we can split data to several subgroups, assign them to different computers to compute the MDS, and then collect the results and combine them into one. In the cell cycle example, we showed that the clustering results of dimension reduction are more stable than the results in the original space. Hence, SC-MDS has overcome the curse of dimensionality in MDS.

## Availability and requirements

• Project name: SCMDS

• Project home page: 

• Operating system(s): OS Portable (Source code to work with many OS systems)

• Programming language: Matlab 6.0 or higher

• Licence: GNU GPL

• Any restrictions to use by non-academics: License needed. Commercial users should contact jengnan@gmail.com

## Authors' contributions

WH Li was the project leader in this study and he investigated the biological issues. HHS Lu and J Tzeng designed and gave the mathematical proof of the fast algorithm of MDS. J Tzeng carried out the programming, data collection and analysis. All authors read and approved the final manuscript.

## Supplementary Material

Additional file 1 Function annotation of original space clustering. This file provided the function annotation of the best K-means clustering result in data presented by original space. Each column refers to a cluster. If the cluster has no efficient annotated function, then the corresponding column is empty. Click here for file

Additional file 2 Function annotation of reduced space clustering. This file provided the function annotation of the best K-means clustering result in data presented by reduced 7-dimensional space. Each column refers to a cluster. If the cluster has no efficient annotated function, then the corresponding column is empty. Click here for file
